# SYSTEMATIC REVIEW AND GAP ANALYSIS ON ALZHEIMER’S DISEASE IN ASIAN AMERICANS, NATIVE HAWAIIANS, AND PACIFIC ISLANDERS

**DOI:** 10.1093/geroni/igz038.431

**Published:** 2019-11-08

**Authors:** Sahnah Lim, Timothy Roberts, Jazmine Wong, Sadia Mohaimin, Young-Jin Sohn, Ragavan Sivanesathurai, Simona C Kwon, Chau Trinh-Shevrin

**Affiliations:** NYU School of Medicine, New York, New York, United States

## Abstract

Background: The Asian American, Native Hawaiian, and Pacific Islander (AANHPI) aging population is rapidly growing and the burden of Alzheimer’s disease and its related dementias (AD/ADRD) will likely mirror this demographic growth. AANHPIs face significant barriers in obtaining timely AD/ADRD diagnosis and services; yet little is known about AD/ADRD in this population. The study objective is to conduct a systematic review on the published literature on AD/ADRD among AANHPIs to identify gaps and priorities to inform future research and action plans. Methods: The systematic review was conducted following the PRISMA Protocol for Systematic Reviews. Co-author (TR), an experienced Medical Librarian, searched PubMed, EMBASE, PsycINFO, Cochrane Central of Clinical Trials, Ageline and Web of Science for peer-reviewed articles describing AD/ADRD among AANHPIs. The search was not limited by language or publication date. Each citation was reviewed by two trained independent reviewers. Conflicts were resolved through consensus. Results: The title/abstract and full texts of 1,447 unique articles were screened for inclusion, yielding 310 articles for analysis. Major research topics included prevalence, risk factors, comorbidities, interventions and outreach, knowledge/perceptions/attitudes, caregiving, and detection tools. A limited number of studies reported on national data, on NHPI communities generally, and on efficacy of interventions targeting AANHPI communities. Conclusion: To our knowledge, this is the first systematic review on AD/ADRD among AANHPI populations. Our review provides a first step in mapping the extant literature on AD/ADRD among this underserved and under-researched population and will serve as a guide for future research, policy and intervention.

